# Temporal Matching in Endoscopic Images for Remote-Controlled Robotic Surgery

**DOI:** 10.1155/2009/627625

**Published:** 2009-04-15

**Authors:** Jia Gu, Rolf Wolters, Ulf Gustafsson

**Affiliations:** ^1^Key Laboratory for Biomedical Informatics and Health Engineering, Shenzhen Institute of Advanced Technology, Chinese Academy of Sciences, Shenzhen 518067, China; ^2^STI Medical Systems, 733 Bishop Street no. 3100, Honolulu, HI 96813, USA

## Abstract

Temporal matching is applied in the frame of the formation of high-level entities in remote-controlled robotic surgery. The objective is to track tumor boundaries over time to improve the segmentation stage in each image of the sequence to facilitate the tracking and localization of the tumor. It makes use of an attributed string matching technique to find the correspondence between tumor boundaries over time. Relationships are then exploited to reconstitute the tumor boundaries and remove the inconsistencies coming from the detection errors. Input data are free form shapes of different length representing the tumor boundary, extracted at a previous stage.

## 1. Introduction

With recent advances in telemedicine technology, it is now possible to perform closed-abdomen surgery on a moving organ of a remote patient using robotic instrument to minimize patient trauma and certain side effects. For robotic assisted surgery, dexterity is enhanced by microprocessor controlled mechanical wrists, which allow motion scaling for reducing gross hand movements and the performance of microscale tasks that are otherwise not possible. So far, two commercially available master-slave manipulator devices are specifically designed for robotic cardiac surgery [1]. Both systems improve the ergonomics of laparoscopic surgery and provide high dexterity, precision, and 3D visualization of the operating field. One of the significant challenges of the in-vivo surgery is the destabilization introduced by respiratory motion, thus severely affecting precise instrument-tissue interactions and the execution of complex grafts. The other one is the precise localization of the tumor boundary, while most of the automatic image-guided algorithms yield incomplete, discontinuous, imprecise detection results. And the two above challenges always trigger each other and form a chick-egg problem. Thus in this paper, we intend to solve this problem by a novel high-level entities formation method.

Many existing methods have been developed to extract structural features [2–4] but, at the same time, included some errors (incomplete detection leading to discontinuities, imprecise localization of contours) that increased the difficulties of the further processing like tracking or 3D reconstruction. One way to overcome these difficulties has been, these last years, to incorporate some interactivity [5]. The contour detection makes use of an interactive procedure for the first image, and is then used as initial model of a snake to extract the contours in the other images of the sequence. Thus in [5], for each model curve, control points were interactively defined as the anchor points of an active contour modeling. Initial control points were interactively selected and the 2D contours represented as a B-spline. The active contour model was then applied to fit each curves of the selected contours. In [5], the segmentation of the first image made use of an interactive tool based on the mouse moving. The operator selected an initial point inside the image, and uncoiled a thread with the mouse, roughly following the boundary. An optimal path research procedure based on a dynamic programming algorithm and using a maximum multiscale response map for constraining the path search was performed between the initial point and the current position of the mouse. The contour was thus piecewise built, by successively moving the mouse along the boundary. 

We focus here on the intermediate level between the segmentation and the reconstruction stages. We look for improving the automatic segmentation result in each image of the sequence by reconstituting high level entities, that is, the shape of tumors, in order to facilitate the tracking and localization of the tumor. A first intraimage analysis was previously performed to detect contours in each image of the sequence. It aimed at forming correspondences from previously extracted contours by using a string matching process [6]. The present work represents a continuation of the previous one. It aims at tracking boundaries over time to establish correspondences in the sequence. These correspondences will then be used to spatially prolong the segments in order to reconstitute the boundaries and get a symbolic description of the structures in the 2D space. We present in this paper the temporal matching method, which is based on an attributed string matching technique. The outline of this paper is as follows. Section 2 describes the temporal procedure. Section 3 provides results on real data. Some prospects for future work are then proposed in conclusion.

## 2. Temporal Matching

Attributed string matching techniques have been broadly applied in pattern recognition with the aim to look for specific shapes, comparing unknown figures occurring in the input image with a set of prototypic models in a classification purpose or emphasizing “landmarks” subsets common to a set of features. More recently, interest was found in image analysis in particular in shape recognition [7–9] and registration in the frame, for instance, of the 3D reconstruction from stereo views [10].

### 2.1. String Matching Concepts

String matching techniques allow measuring the similarity degree between two strings of symbols. 

Let *S*_*n*_ ∈ Σ^*l*_*n*_^ be an arbitrary string of length *l*_*n*_ = |*S*_*n*_| defined on a discrete alphabet Σ. We denote *s*_*n*_^*i*^ the *i*th symbol of *S*_*n*_ for *i* ∈ {1 ⋯ |*S*_*n*_|} ∈ such as *S*_*n*_^*l*_*n*_^ = *s*_*n*_^0^ *s*_*n*_^1^ ⋯ *s*_*n*_^*i*^ ⋯ *s*_*n*_^*l*_*n*−1_^. An edit distance, based on edit operations (insertion, deletion, and substitution of symbols), is then defined as the minimum cost operation sequence over all possible sequences of operations *O* (*O* = *o*_1_ *o*_2_ ⋯ *o*_*m*_) with *o*_*k*_ equals to one of the three above cited edit operations. It makes use of a cost matrix, which specifies the costs *γ*(*o*_*k*_) of individual elementary operations for all combination of symbols. The total cost of the transformation is then calculated by summing up the elementary costs applied to each operation in the sequence. The distance associated with the minimum cost operation sequence is given by the relation:

(1) $$\delta (S_{n}^{l_{n}},S_{p}^{l_{p}})=\left\{\frac{{\text{Min}}_{O}\left(\Gamma_{s_{n,s_{p}}}\left(O\right)\right)}{O}:S_{n}^{l_{n}}\to S_{p}^{l_{p}}\right\},$$

with Γ_*s*_*n*_,*s*_*p*__(*O*) = ∑_*v* = 1_^*m*^ *γ*(*o*_*v*_).

Its computation can be carried out using optimization techniques. Among these ones, the classical dynamic programming, initially designed by Wagner & Fisher [6], has largely been used to calculate the edit distance. 

The process goes through the edit matrix computation *D*(*i*, *j*), 1 ≤ *i* ≤ *l*_*n*_, 1 ≤ *j* ≤ *l*_*p*_ using the following recursive relation:

(2) $$\begin{array}{ll} D\left(i,j\right) & =\min\;\{(D(i-1,j-1)+\gamma (s_{n}^{i}\to s_{p}^{j})),\\ & \;\;\;\;\;(D(i-1,j)+\gamma (s_{n}^{i}\to \lambda )),\\ & \;\;\;\;\;(D(i,j-1)+\gamma (\lambda \to s_{p}^{j}))\}, \end{array}$$

where *λ* denote the empty symbol and *γ*(*s*_*n*_^*i*^ → *s*_*p*_^*j*^), *γ*(*λ* → *s*_*p*_^*j*^), *γ*(*s*_*n*_^*i*^ → *λ*), respectively, characterize the substitution, insertion, and deletion costs. The least cost operation sequence is obtained through a search of a least cost path in the graph. The result is a sequence of ordered pairs of positions in *S*_*n*_^*l*_*n*_^ and *S*_*p*_^*l*_*p*_^ corresponding to substitutions. With a least-cost trace from *S*_*n*_^*l*_*n*_^ to *S*_*p*_^*l*_*p*_^, *T*(*S*_*n*_^*l*_*n*_^, *S*_*p*_^*lp*^) is hence defined as the set of ordered pairs of integer (*i*, *j*) corresponding to the indices of the paired symbols between the two strings.

### 2.2. Shape Representation and Cost Function

Let *L*_*t*,*n*_ and *L*_*t*+*d*,*m*_ two lines to be matched with (*t*, *t* + *d*) characterizing the image number in the sequence and (*n*, *m*) the line numbers in each image. Elements of *L*_*t*,*n*_ and *L*_*t*+*d*,*m*_ were, respectively, noted *p*_*t*,*n*_^*i*^ and *p*_*t* + *d*,*m*_^*j*^, *i* and *j* being the element indexes for each line. Four attributes were associated to each pixel *p*_*t*,*n*_^*i*^: the intensity *I*(*p*_*t*,*n*_^*i*^), the Euclidean location ${\overset{\to }{d}}_{t,n}^{i},$ the tangent ${\overset{\to }{t}}_{t,n}^{i},$ and the local curvature *κ*_*t*,*n*_^*i*^. Each line was thus expressed as a sequence *S*_*t*,*n*_^*l*_*n*_^ of symbols (*l*_*n*_ being the length of the string), each symbol corresponding to a set of attributes, we formalized as a vector $s_{t,n}^{i}=\left(I\left(p_{t,n}^{i}\right),{\overset{\to }{d}}_{t,n}^{i},{\overset{\to }{t}}_{t,n}^{i},\kappa_{t,n}^{i}\right).$ We designed a multiparametric cost function between sets of two vectors (*s*_*t*,*n*_^*i*^, *s*_*t* + *d*,*m*_^*j*^) based on perceptual criteria: parallelism, proximity, curvature, and intensity similarity.

Deletion and insertion costs were set to 0.5, while the substitution cost, defined by the function *f *(*s*_*t*,*n*_^*i*^, *s*_*t* + *d*,*m*_^*j*^), was expressed on the [0, 1] interval:

(3) $$\begin{array}{ll} f(s_{t,n}^{i},s_{t+d,m}^{j}) & =\alpha \cdot f_{i}\left(I\left(p_{t,n}^{i}\right),I\left(p_{t+d,m}^{j}\right)\right)+\beta \cdot f_{p}({\overset{\to }{t}}_{t,n}^{i},{\overset{\to }{t}}_{t+d,m}^{j})\\ & \;+\lambda \cdot f_{\kappa }\left(\kappa_{t,n}^{i},\kappa_{t+d,m}^{j}\right)+\gamma \cdot f_{d}(p_{t,n}^{i},p_{t+d,m}^{j}), \end{array}$$

where *α*, *β*, *λ*, *γ* were weighting coefficients such that *α* + *β* + *λ* + *γ* = 1. 

Each function was defined as an exponential of the form *f *(, ) = *e* ^−((Δ_max_−|Δ|)/|Δ|)^, where Δ_max_ characterized the maximum permitted deviation between two symbols for a given criterion and Δ, the difference measured between the two considered symbols. Δ_max_ definition depends on the limit, we set up for each parameter deviation so that 0 ≤ *f *(, ) ≤ 1, value 0 depicting a good shape similarity.

### 2.3. Tumor Boundary Formation

This stage aimed at establishing the temporal correspondences between segments of contours along the sequence. The attributed string matching was applied on sets of pairewise images, all possible temporal associations being taken into consideration. 

For each contours *L*_*t*,*n*_ in image *I*_*t*_, a contours neighborhood *V*_(*t*,*t*+*d*),*n*_ was built in image *I*_*t*+*d*_ such as

(4) $$V_{(t,t+d),n}=\{\frac{L_{t+d,m}}{m}=1\cdots N_{n},\;\text{distance}(L_{t,n},L_{t+d,m})<\Delta d_{\text{max}}\},$$

where Δ*d*_max_ sets the upper limit for the contour displacement between two instants *t* and *t* + *d* and *N*_*n*_ denotes the cardinal of *V*_(*t*,*t*+*d*),*n*_.

All the possible combinations of contours, inside this neighborhood, were then considered for the pairing. With the segment running direction for the matching being not prior known, each extremity of a segment was numbered and two runs were considered according to these extremities. Two associations were then considered among the four possible combinations {(*L*_*t*,*n*_^1^, *L*_*t* + *d*,*m*_^1^), (*L*_*t*,*n*_^1^, *L*_*t* + *d*,*m*_^2^)}, exponent 1 and 2 representing the extremity number. Attributed strings (*S*_*t*,*n*_^*l*_*n*_^, *S*_*t* + *d*,*m*_^*l*_*m*_^) were then assigned to each two lines (*L*_*t*,*n*_, *L*_*t*+*d*,*m*_). The attributed string matching process was applied for each two pairs of lines and the right running direction corresponded to the best association among the two, when choosing the minimal edit distance as selection criterion.

Because multiple assignments can occur in some situations (when several contours are close to each other or when spurious segments are present), two criteria were thus defined in order to keep only the one-to-one matching

1. A threshold on the normalized edit distance aimed to remove all the inconsistent associations: matched lines whose edit distance was greater than this threshold were cancelled.
2. An overlapping criterion was then considered to remove redundant associations. These associations are considered as right if there is no overlapping between the matched sets. On the contrary, these associations are wrong and have to be filtered. Thus when several candidate curves were associated to the same curve with some overlapping between these associations, the matched lines were sorted out on the edit distance value and the established trace between the paired symbols. Considering for each line *L*_*t*,*n*_, the matched line list by decreasing edit distance values, if the trace between the matched pixels intersected a curve, the pair of lines having the highest edit distance value was removed.

## 3. Result and Discussion

**Figure 1:**
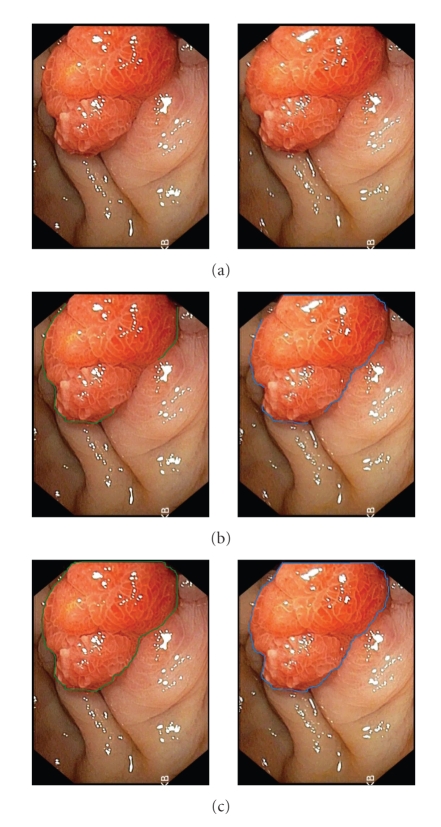
String matching process and boundary formation on two successive images of a sequence: (a) original acquired images from endoscope, (b) initial contour extraction, (c) boundary formation: the discontinuities have been reconstituted.

The matching procedure was applied to endoscopic images acquired from standard optical colonoscope (from our collaborator: Stanford University), and the preliminary results can be found in Figure 1. Whatever the detection operator and the decision rule, it must be accepted that no exact result can be derived. This statement, which applies to edge detection too, was the first motivation of this work. In a first stage, edges were grouped using spatial properties to construct coherent pieces [6]. This process was operated in an intraimage mode. The next stage, that is, the temporal matching, takes advantage of the motion correspondence to solve spatial ambiguities and improve the segmentation results in presence of occlusion or discontinuities on the images. The matching process was led over time along the sequence. It was pairewise applied on the contour and allows at the same time to get a first approximation of the motion between the structures. The parameters setting resulted from a learning stage, which allowed studying the behavior of the algorithm according to the parameters choice and combination. This study showed that the results were not sensitive to the choice of the parameters. The best result was obtained with the following setting: *α* = 20%, *β* = 15%, *λ* = 30%, *γ* = 35%, Δ*I*_max_ = 20 (maximum intensity difference), Δ*θ*_max_ = *π*/4 (angular difference between the tangents), Δ*κ*_max_ = 0.5 (maximum curvature with |Δ*κ*| = |*κ*_*t*, *n*_^*i*^ − *κ*_*t* + *d*,*m*_^*j*^|/8), Δ*d*_max_ = 30 (maximum distance). The distance parameter role is to penalize the structure displacements beyond this value. For these parameters, we computed the mean cross-correlation, the mean distance, and the mean normalized edit distance over the set of matched lines. We obtained the respective values: 0.963, 3.121, and 0.275. The branches formation was then performed over the sequence, based on the matching process results.

## 4. Conclusion

In this paper, we made use of an attributed string matching technique for the formation of high-level entities like boundaries of tumors in endoscopic images for remote-controlled robotic surgery. The advantage of the method is that few parameters have to be set to track the contours over time. The established temporal relationships were exploited to spatially locate the contours and reconstitute the tumor boundaries. These results will be further used to complete the tumor boundaries that were previously performed on each image of the sequence [6]. This work represents a contribution to the difficult problem of the tumor tracking and localization in robot-assisted minimal invasive surgery.
